# Endotracheal tube-mounted camera-assisted intubation versus conventional intubation in intensive care: a prospective, randomised trial (VivaITN)

**DOI:** 10.1186/s13054-018-2152-4

**Published:** 2018-09-22

**Authors:** Jörn Grensemann, Lars Eichler, Nuowei Wang, Dominik Jarczak, Marcel Simon, Stefan Kluge

**Affiliations:** 1 Department of Intensive Care Medicine, University Medical Centre Hamburg-Eppendorf, Martinistraße 52, 20246 Hamburg, Germany; 2 Department of Respiratory Medicine, University Medical Centre Hamburg-Eppendorf, Martinistraße 52, 20246 Hamburg, Germany

**Keywords:** Airway management D058109, Critical care D003422, Diagnostic techniques, Respiratory system D003948, Intubation, Intratracheal D007442, Respiration, Artificial D012121

## Abstract

**Background:**

For critically ill patients, effective airway management with a high first-attempt success rate for endotracheal intubation is essential to prevent hypoxic complications during securing of the airway. Video guidance may improve first-attempt success rate over direct laryngoscopy (DL).

**Methods:**

With ethics approval, this randomised controlled trial involved 54 critically ill patients who received endotracheal intubation using a tube with an integrated video camera (VivaSight™-SL tube, VST, ETView Ltd., Misgav, Israel) or using conventional intubation under DL.

**Results:**

The two groups did not differ in terms of intubation conditions. The first-attempt success rate was VST 96% vs. DL 93% (not statistically significant (n. s.)). When intubation at first attempt failed, it was successful in the second attempt in all patients. There was no difference in the median average time to intubation (VST 34 s (interquartile range 28–39) vs. DL 35 s (28–40), n. s.). Neither vomiting nor aspiration or accidental oesophageal intubation were observed in either group. The lowest pulsoxymetric oxygen saturation for VST was 96 (82–99) % vs. 99 (95–100) % for DL (n. s.). Hypotension defined as systolic blood pressure < 70 mmHg occurred in the VST group at 20% vs. the DL group at 15% (n. s.).

**Conclusion:**

In this pilot study, no advantage was shown for the VST. The VST should be examined further to identify patient groups that could benefit from intubation with the VST, that is, patients with difficult airway conditions.

**Trial registration:**

ClinicalTrials.gov, NCT02837055. Registered on 13 June 2016.

## Background

In comparison with patients undergoing elective surgery, critically ill patients requiring endotracheal intubation are at increased risk of life-threatening complications, mostly related to hypoxaemia during airway management [1]. During endotracheal intubation, expedited airway management reduces the rate of complications by shortening the time from the onset of anaesthesia-induced apnoea to the establishment of an airway and the beginning of artificial ventilation. However, in critically ill patients this is complicated by an increased incidence of difficult airways that is estimated at 10% [2, 3] and is approximately twice as high as in patients undergoing elective surgery [4]. Normally, endotracheal intubation is facilitated by direct laryngoscopy (DL) but this approach may fail in patients with difficult airway conditions increasing the rate of life-threatening complications from approximately 30 to 50% [5].

Because of the high incidence of airway-related complications, several methods have been proposed to improve the safety and reliability of endotracheal intubation in intensive care medicine. One of these methods is videolaryngoscopy (VL) [6]. With this technique, the larynx is visualised indirectly by a camera or a fibreoptic system. Although initial data have pointed to an increase in the first-attempt success rate [7, 8], this finding was challenged by the latest meta-analyses indicating no difference when used for unselected intubations in intensive care [9, 10].

Recently, an endotracheal tube with an integrated camera at its tip (VST) has been introduced that permits continuous visualisation of the tube’s position on a monitor connected to the camera (VivaSight™-SL, ETView Ltd., Misgav, Israel) [11]. The camera is laminated onto the anterior wall of the tube. In theory, the intubation with the advancement of the endotracheal tube through the vocal chords into the trachea should be visible on the connected monitor. We hypothesised that endotracheal intubation may be improved with this camera-aided intubation and that the tube placement could be confirmed immediately on the monitor by visualisation of tracheal cartilages. We assessed the first-attempt success rate and the number of attempts to achieve successful intubation against DL in a prospective, randomised, controlled study.

## Methods

### Study design

The VivaITN trial was a prospective randomised study conducted in the Department of Intensive Care Medicine at the University Medical Centre, Hamburg-Eppendorf. Patients were eligible if they were at least 18 years old, received endotracheal intubation for a clinical indication, and written informed consent was obtained from the patient or a legal guardian. We sought to enrol 54 patients who were randomised (using sealed, opaque envelopes) in a 1:1 ratio to intubation with the VST or with the conventional method of DL. We included patients receiving urgent endotracheal intubation defined as intubation in the setting of acute respiratory failure or elective intubation defined as an intubation performed solely for the purpose of ventilatory support and airway protection during a procedure [12]. An a priori power analysis indicated that a sample size of 54 would be sufficient to detect a difference of 35% in the first-attempt success rate with error probabilities of α = 0.05 and 1-β = 0.80 (PASS version 08.0.6, NCSS, LLC. Kaysville, UT, USA). The study was registered on ClinicalTrials.gov (NCT02837055).

### Patients’ characteristics

Demographic data were obtained from the patients’ electronic records (Integrated Care Manager ICM, version 8.12, Drägerwerk, Lübeck, Germany). The Acute Physiology And Chronic Health Evaluation II (APACHE II) score [13] and the Sequential Organ Failure Assessment (SOFA) score [14] were recorded on the day of examination as measures of disease severity.

### Assessment of difficulty of intubation

To assess the difficulty of intubation, we obtained the thyromental distance, mouth opening and the Mallampati Score [15]. Patients were examined with respect to anatomical conditions which are associated with difficult intubation, that is, restricted mobility of the cervical spine, retrognathia, and obesity.

### Intubation

Endotracheal tubes with an inner diameter of 7.5 mm for female patients or 8.0 mm for male patients were chosen. In patients randomised to the VST group, the tube-mounted camera was connected to a VivaSight™-Max monitor (ETView Ltd., Misgav, Israel), which was attached to the bed rails. Patients were anaesthetised with propofol and sufentanil. Rocuronium was used for muscle relaxation. DL was performed irrespective of the randomisation, and the view of the larynx was assessed according to the method of Cormack and Lehane [16]. Camera-assisted intubation was commenced in patients randomised to the VST group. Patients randomised to the conventional group were intubated with conventional DL.

### Outcome parameters

The primary outcome parameters were the first-attempt success rate and the total number of attempts at successful intubation. Secondary outcome parameters were the time to successful intubation, the time to successful intubation with one attempt, the average number of attempts at intubation, vomiting or aspiration during intubation, accidental oesophageal intubation, a decrease of the oxygen saturation by pulse oximetry below 80%, and hypotension defined as systolic blood pressure below 70 mmHg (Infinity Delta vital signs monitor, Drägerwerk, Lübeck, Germany). Time to successful intubation was measured from the removal of the patient’s face mask for preoxygenation to the first definite sign of successful intubation, defined as visualisation of tracheal cartilages with the VST camera or continuous positive end-tidal carbon dioxide reading (at least three breaths without a significant visual decrease in capnography).

### Statistics

Microsoft Excel 2016 (Microsoft Corp., Redmond, WA, USA) was used for data management and the SPSS statistical software package (version 23, IBM Inc., Armonk, NY, USA) was used for statistical analysis. We used the *t* test and U test for comparisons of parameters as applicable and contingency tables with the chi-square test and Fisher’s test. Two-tailed *P* values < 0.05 were regarded as statistically significant. Data are given as median and interquartile range.

## Results

**Fig. 1:**
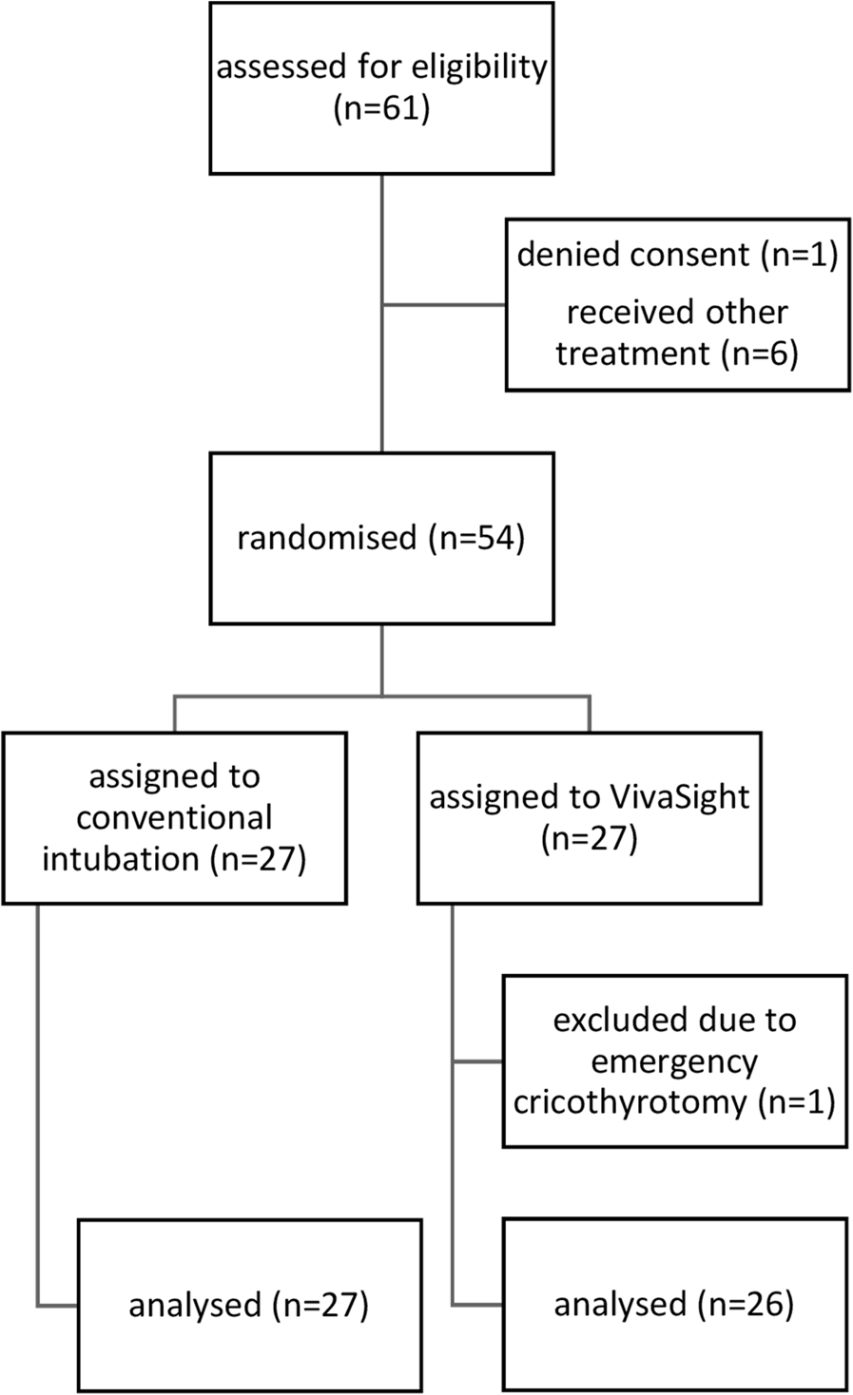
Consolidated standards of reporting trials (CONSORT) diagram

From June 2016 to January 2018, a total of 54 patients receiving endotracheal intubation were randomised to either camera-assisted intubation with the VST or conventional intubation under DL in a 1:1 ratio (see Fig. 1). Patients’ baseline characteristics are shown in Table 1. No patient presented with an anatomical condition likely to be associated with difficult intubation. Patients were intubated by attending physicians or fellows. The median professional experience was 14 years (14–15) in the VST group and 14 years (7–14) in the DL group. Intubation conditions were similar between groups (see Table 2). One patient in the VST group was excluded due to a *cannot ventilate – cannot intubate* situation caused by severe laryngeal oedema, and received emergency cricothyrotomy.

**Table 1:** Patient characteristics

	VivaSight (*n* = 26)	Conventional intubation (*n* = 27)
Age (years)	63 ± 15	57 ± 14
Gender	Male: 58% Female: 42%	Male: 52% Female: 48%
SOFA score	6 ± 4	6 ± 4
APACHE II score	16 ± 7	18 ± 6

Data are shown as mean ± standard deviation

*SOFA* Sequential Organ Failure Assessment, *APACHE II* Acute Physiology And Chronic Health Evaluation II

**Table 2:** Intubation conditions

	VivaSight (*n* = 26)	Conventional intubation (*n* = 27)	*P*
Mallampati grade	1: 23%	1: 15%	n.s.
	2: 62%	2: 70%	
	3: 15%	3: 15%	
	4: 0%	4: 0%	
Thyromental distance (cm)	5.8 ± 1.0	5.7 ± 1.3	n.s.
Maximum mouth opening (cm)	4.5 (3.6–5.5)	4.5 (4.0–5.0)	n.s.
Cormack and Lehane grade	1: 85%	1: 74%	n.s.
	2: 11%	2: 26%	
	3: 4%	3: 0%	
	4: 0%	4: 0%	

Data are shown as mean ± standard deviation or median and interquartile range, as appropriate. *n.s.* not statistically significant

The first-attempt success rate was 96% in the VST group and 93% in the conventional group (not statistically significant (n.s.) with one patient in the VST group and two patients in the conventional group requiring a second intubation attempt (average number of attempts 1.0 vs. 1.1, n.s.). An overview of the outcome parameters is given in Table 3. The mean procedure duration from mouth opening to confirmed intubation was 34 s (28–39) in the VST and 35 s (28–40) in the conventional group (n.s.). Time to successful intubation on the first attempt, the rate of hypoxia, and the rate of arterial hypotension did not differ between the groups. Vomiting or aspiration did not occur in any patient. In 13 of 26 patients in the VST group, the camera image became blurred during intubation due to lens contamination by secretions, thus substantially hindering intubation by camera guidance.

**Table 3:** Outcome parameters

	VivaSight	Conventional intubation	*P*
First-attempt success rate	96%	93%	n.s.
Total number of attempts	1 attempt: 25 (96%) 2 attempts: 1 (4%)	1 attempt: 25 (93%) 2 attempts: 2 (7%)	n.s.
Average number of attempts	1.0	1.1	n.s.
Time to successful intubation (s)	34 (28–39)	35 (28–40)	n.s.
Time to successful intubation with one attempt (s)	33 (28–39)	33 (28–38)	n.s.
Vomiting/aspiration during intubation	None	None	n.s.
Accidental oesophageal intubation	None	None	n.s.
SpO_2_ < 80% SpO_2_ (%)	19% 96 (82–99)	15% 99 (95–100)	n.s.
Systolic blood pressure < 70 mmHg Systolic blood pressure (mmHg)	20% 93 ± 27	15% 95 ± 25	n.s.

Data are shown as mean ± standard deviation or median and interquartile range, as appropriate. *n.s.* not statistically significant, *SpO*_*2*_ pulse arterial oxygen saturation

## Discussion

In this prospective randomised trial comparing endotracheal intubation assisted by a tube-mounted camera (VST) with intubation by DL, we found no difference in the first-attempt success rate or the average number of attempts at successful intubation. Concerning other tested variables, no difference was detected between the two groups.

Thus far, the VST has shown promising results for endotracheal intubation via supraglottic airway devices [11, 17], in mannikins [18–22] and in a cadaver study [23]. Furthermore, the guidance of percutaneous dilatational tracheostomy by VST has been evaluated and shown to be feasible [24, 25].

In critically ill patients, airway management is a hazardous procedure due to the high rate of patients with respiratory insufficiency and pre-existing hypoxaemia. Accordingly, the most prevalent complications during endotracheal intubation in the intensive care unit are hypoxaemic complications in approximately 30% of patients receiving endotracheal intubation, with 2% of patients even requiring cardio-pulmonary resuscitation [26, 27]. Because multiple intubation attempts are independently associated with complications [28], it is crucial to increase the first-attempt success rate to prevent complications.

The first-attempt success rate of intubation depends on physicians’ experience and increases with physicians’ training [29–31]. In our study, patients were intubated by attending physicians or fellows with several years of experience in airway management. Further, in our study, the first-attempt success rate was above 90% in both groups, which is congruent to data showing the first-attempt success rate for experienced physicians to be approximately 90% [30, 32]. However, many intubations in intensive care are performed by residents with less experience than the physicians in our study [30]. Therefore, our data may not be representative. Because mannikin studies with inexperienced operators have shown a benefit for the VST over DL with a higher success rate [18, 19], we cannot exclude the possibility that the VST performs better in the hands of inexperienced operators. However, this might not be a proof of VST being superior but a sign of lower quality DL in the hands of inexperienced operators. Nevertheless, in this case, the VST might be an alternative to conventional DL in resource-limited settings with the unavailability of a physician experienced in airway management; however, in our opinion this approach should only be chosen as the last option.

Videolaryngoscopy (VL) has been widely used for intubation in intensive care. It had been hypothesised that the first-attempt success rate could be increased over DL, but the latest data have shown no benefit when used in an unselected cohort of intensive care patients [9] and even inferiority when used pre-clinically [10]. When analysing patients in whom there was an expectation of difficulty in achieving intubation due to the condition of the airways, VL led to fewer failed intubations than DL. A potential benefit of VST in the hands of experienced operators managing anatomically difficult airways may be hypothesised. However, a direct comparison with alternative instruments such as VL is lacking. Although not an exclusion criterion, no patient in our study was expected to be difficult to intubate due to the anatomy of the airway and in analogy with VL, we did not detect any difference in the first-attempt success rate.

The procedure duration was identical in both groups. An intubation duration of approximately 30 s has been recorded for experienced anaesthesiologists using the VST and DL [8, 33, 34], although one study identified a difference of 5 s in favour of the VST [33]. On the other hand, the use of VL could prolong the time to intubation [8, 34]. Regarding our data and previously published data [33], there is no indication that intubation with the VST prolongs the duration of intubation. This may be beneficial in patients who require expedited airway management due to respiratory insufficiency with a diminished oxygen reserve and apnoea tolerance. Moreover, the usefulness of the device may increase with the passage of an individual learning curve. This is a factor that makes comparison of newly established methods with routinely applied standard methods difficult.

In our study, camera-guided intubation was substantially hindered in every second patient due to a contamination of the lens by secretions. The VST is equipped with a rinsing channel to clear the lens from secretions but in all cases, endotracheal intubation was completed by DL in favour of expedited establishment of the airway. The problem of a soiled airway is common in intensive care patients as opposed to mannikins and cadavers in studies in which the VST was evaluated for endotracheal intubation. For one VL model, severe lens contamination (defined as impossibility to visualise the larynx) occurred in 3.2% [35], while the same group of authors found an incidence of severe lens contamination for the same VL model in 0.5% of clean airways and in 1.3% of soiled airways [36]. In the latter study, the first-attempt success rate by VL dropped from approximately 90% in clean airways to approximately 80% in soiled airways compared with 75% to 65% by DL. As opposed to most videolaryngoscopes where the camera is recessed and therefore partially protected against secretions, the camera at the tip of the VST seems more vulnerable to lens contamination, decreasing its usefulness in the guidance of endotracheal intubation.

Our study has certain limitations. Not being a blinded study, the types of intervention compared were at risk of ascertainment bias, though quick and atraumatic intubation was the set goal in both groups. The first-attempt success rate was higher than anticipated and therefore the sample size may not have been sufficient to detect a difference between the groups. Due to lens contamination, half of the patients randomised to the VST group basically received conventional intubation, highlighting the decreased usefulness of VST in patients with a high incidence of soiled airways. Therefore, our sample size is possibly not large enough to detect significant differences between the two groups. Because of the requirement to obtain informed consent, only patients receiving elective or urgent intubation could be included. Therefore, no data are available on the performance for emergency airway management. None of the patients who underwent randomisation had a difficult airway and it would be of interest to establish whether the VST would be beneficial under these circumstances.

## Conclusion

In patients requiring elective or urgent endotracheal intubation in the intensive care unit, no additional benefit was demonstrated for the VST over DL. The performance of the VST in patients with an expected difficult airway should be evaluated separately. Airways soiled with secretions may reduce the usability of the VST due to lens contamination. Because of a high first-attempt success rate in both groups, we suggest that the VST should be evaluated in a larger cohort of patients and should be evaluated when applied by physicians with less expertise in intubation.

## Abbreviations

DL: Direct laryngoscopy
n.s.: Not statistically significant
VL: Video laryngoscopy
VST: VivaSight™-SL endotracheal tube
